# The impact of N‐acetylcysteine on lactate, biomarkers of oxidative stress, immune response, and muscle damage: A systematic review and meta‐analysis

**DOI:** 10.1111/jcmm.70198

**Published:** 2024-12-04

**Authors:** Marcin Sadowski, Emilia Zawieja, Agata Chmurzynska

**Affiliations:** ^1^ Department of Human Nutrition and Dietetics Poznań University of Life Sciences Poznań Poland

**Keywords:** glutathione, interleukin 6, muscle damage, muscle soreness, N‐acetylcysteine

## Abstract

N‐acetylcysteine (NAC) is a compound whose mechanism of action is intricately linked to the provision of cysteine for glutathione synthesis. It has been used in medicine and has also made significant inroads into sports, as it can modify the levels of several biomarkers, including those of oxidative processes, inflammation and muscle damage after exercise. Because the effectiveness of NAC supplementation is unclear, the primary objective of the present study was to perform a meta‐analysis elucidating how NAC supplementation alters the concentrations of GSH (glutathione), GSSG (glutathione disulfide), TBARS (thiobarbituric acid reactive substances), IL‐6 (interleukin 6), TNF‐α (tumour necrosis factor alpha), CK (creatine kinase), lactate, and muscle soreness after physical exertion. Suitable studies were searched for from February to September 2023, and the results of those included (*n* = 20) indicate that NAC supplementation significantly diminishes both muscle soreness (*p* = 0.03; the mean difference (MD) of NAC's effect was −0.43 with a 95% confidence interval (CI), −0.81, −0.04) and lactate concentrations after exercise (*p* = 0.03; the MD −0.56 mmol/L; 95% CI, −1.07, −0.06). A substantial decrease was observed in concentrations of IL‐6 (*p* = 0.03; the standardized MD (SMD) was −1.71; 95% CI, −3.26, −0.16) and TBARS (*p* = 0.02; SMD was −1.03, 95% CI, −1.90, −0.15). Furthermore, an elevation in GSH concentration was observed following supplementation. However, we saw no significant effect of NAC on TNF‐α, CK or GSSG concentrations. NAC supplementation holds promise for attenuating muscle soreness, lactate, TBARS and IL‐6 concentrations and increasing GSH level following physical exertion.

## 1 INTRODUCTION

Research into the effects of N‐acetylcysteine (NAC) on oxidative stress, immune response and muscle damage biomarkers has been motivated by the proposed role of this substance in cellular redox balance and immunomodulation.^1^, ^2^, ^3^ Nevertheless, a recent systematic review concluded that more research into the effects of NAC on immune response and muscle damage biomarkers is needed.^4^ Similarly, the Australian Institute of Sport considers NAC to have promising results for sports nutrition, but that more research is necessary to assess its effectiveness.

NAC serves as a precursor to glutathione (GSH), which is a key antioxidant involved in removing ROS and maintaining cellular integrity.^5^ This antioxidative capability suggests that NAC may play a crucial role in counteracting the oxidative stress associated with various pathological conditions.^6^ The production of ROS increases during intense exercise. To maintain cellular integrity, GSH serves as a substrate for the antioxidant enzyme glutathione peroxidase (GPx), which neutralizes ROS. GSH acts also as a direct scavenger that neutralizes ROS, undergoing oxidation to form glutathione disulfide (GSSG).^7^ Depletion of intracellular GSH levels can result in the excessive accumulation of ROS, the formation of lipid peroxidation products (such as thiobarbituric acid reactive substances–TBARS), cellular damage and inflammation.^8^

Furthermore, the potential immunomodulatory properties of NAC has attracted attention on account due to its suggested capacity to modulate inflammatory processes.^9^ By replenishing intracellular GSH levels, NAC may contribute to regulating the immune response and attenuation of inflammatory states, with implications for conditions characterized by immune dysregulation. The role of GSH in the immune system is related to the major effects of this antioxidant on T lymphocytes, dendritic cell function and neutrophil phagocytosis.^10^ Moreover, the level of GSH in macrophages is associated with the production of Th1/Th2 cytokines.^10^ One of the first stages in antigen degradation is the reduction of disulfide bonds, a process that requires GSH. GSH inhibits the production of proinflammatory cytokines and is necessary in the synthesis of interferon gamma, which in turn is involved in inhibiting the spread of pathogens in the body.^10^

NAC has been investigated in exercise physiology for its role in the fatigue and muscle damage induced by physical exertion.^11^, ^12^, ^13^, ^14^ Exercise‐induced muscle damage is associated with immune response and oxidative stress,^15^, ^16^, ^17^ and the multifaceted mechanisms of NAC make it an interesting candidate for counterbalancing these effects. A cytokine whose production is notably increased after intense exercise is tumour necrosis factor alpha (TNF‐α). TNF‐α is recognized as a pro‐inflammatory cytokine, and its secretion increases as a consequence of cellular damage after intense exercise. For instance, post‐marathon studies have observed a two‐fold increase in TNF‐α levels.^18^ The pro‐inflammatory cytokine IL‐6 is secreted in muscles by T‐cells following intense exercise and is the most familiar interleukin in research into the immune response to exercise. However, increased secretion of IL‐6 is also associated with increased synthesis of anti‐inflammatory interleukin‐10 (IL‐10). Moreover, synthesis of IL‐6 is associated with infection defence, improved lipid metabolism, and glucose metabolism.^19^ Nevertheless, prolonged increases in the levels of this cytokine may lead to inflammation and disturbances in adaptation to exercise.

During exercise, lactate level increase, leading to increased ATP demands. Lactate is also a substrate for gluconeogenesis and serves as am autocrine, paracrine, and endocrine signalling molecule.^20^ Increased lactate levels are associated with the release of hydrogen anions in the muscles and with decreased pH^21^; the latter can lead to more rapid fatigue during exercise.^21^, ^22^ Muscle damage induced by physical exertion often results in delayed muscle soreness, but also to increased levels of creatine kinase (CK). Structural damage and oxidative stress may lead to muscle cell damage and leaking of this enzyme into the bloodstream. Measurement of CK in the bloodstream is thus often used as a biomarker of muscle damage and recovery.^23^

By assessing the effects of NAC on oxidative, inflammatory and muscle damage, this meta‐analysis aims to elucidate whether NAC supplementation can confer benefits in the context of post‐exercise recovery and adaptation. The systematic review and meta‐analysis presented here adopt a meticulous approach in line with established guidelines such as PRISMA (Preferred Reporting Items for Systematic Reviews and Meta‐Analyses).^24^ Our meta‐analysis aims to assess the significance and magnitude of the effects of NAC on the studied parameters. In this way, we hope to provide a consolidated perspective on the effects of NAC and to offer valuable insights for researchers, clinicians, and individuals interested in optimizing health and performance. Our meta‐analysis and systematic review can also help locate gaps in the literature that can be addressed in future to give a more comprehensive assessment of the effectiveness of NAC in sports.

## 2 AIMS

- Assessment of the effects of NAC supplementation on parameters of oxidative stress (GSH, GSSG, TBARS) and immunological parameters (IL‐6, TNF‐α) after exercise.
- Assessment of the effect of NAC supplementation on muscle damage markers (muscle soreness, CK) and lactate after exercise.

## 3 METHODS

### 3.1 Information sources

Search for relevant studies was conducted across electronic databases including Medline, Scopus, and Web of Science, the final search conducted on September 7, 2023. No publication date filter was applied to any of the searches.

### 3.2 Search strategy

The search employed MeSH (Medical Subject Headings), Entry Terms and keywords related to NAC, physical performance, exercise‐induced muscle damage, exercise‐induced inflammation and oxidative biomarkers. Search pattern which was used to search in all databases can be found in Appendix S1. The results were filtered by text availability (free full text or full text), research type (clinical trial or randomized clinical trial) and language (English). The results were additionally restricted to research including adult men and women as a participants. Initial screening focused on the title of the research. Duplicates were removed and the abstracts were reviewed by two authors (MS and EZ). Abstracts that did not meet inclusion criteria were removed from further analysis. Finally, full texts were evaluated in terms of predefined inclusion and exclusion criteria. Initial screening focused on the title of the research.

### 3.3 Inclusion criteria

This systematic review and meta‐analysis included published studies that met the following criteria:

- Written in English.
- Had humans as participants.
- Participants were healthy males and females of all ages and all training experience levels.
- Used a randomized control or cross‐over design.
- NAC was administered (IV or oral) with no restrictions on the timing, dose, form or frequency of administration.
- Effects of maximal performance test and clear performance outcome measures were reported.
- Inflammatory (TNF‐alpha, IL‐6), muscle damage (muscle soreness, CK, lactate) and oxidative markers (GSH, GSSG, TBARS) were reported.
- Adequate information on the baseline and end‐trial studied markers in both control and NAC group was reported.

### 3.4 Exclusion criteria

Animal and in vitro studies, articles in languages other than English, conference abstracts, observational studies, case reports, studies of supplementation combined with additional ingredients and studies that lacked information on the initial and final concentrations of the selected markers were excluded from the survey.

### 3.5 Study selection

Studies found through the search were independently and redundantly qualified for inclusion in the review by two reviewers (MS and EZ) in a standardized manner. Discrepancies between the reviewers were resolved through discussion, and when a consensus could not be reached, a third author (AC) intervened to resolve the dispute.

### 3.6 Data extraction

One author (MS) meticulously analysed the data from the included studies, while the second author (EZ) verified this information. Discrepancies were resolved through discussion between the two reviewers, and in cases of disagreement, the third author (AC) intervened to settle the dispute. The data extraction included details such as authors and publication year, country, study type, sample size, sample characteristics, intervention details (dose, duration), placebo group information and the type of exercise performed. Data from each of included studies was extracted to excel file by first author (MS). After extraction file with collected data was sent to second author (EZ).

Quantitative analysis was used to assess changes in the concentrations of TNF‐α, IL‐6, muscle soreness, lactate, CK, GSH, GSSG and TBARS. Where data were incomplete, the online application Plot Digitizer (https://plotdigitizer.com/app, accessed in September 2023) was used to retrieve data from graphs, or the study authors were contacted to obtain the missing information.

### 3.7 Sensitivity analysis

Sensitivity analysis was conducted for each of the parameters analysed in the meta‐analysis. The impact of individual study results on changes in the analysed parameters related to the effect of NAC supplementation was assessed based on changes in statistical significance.

### 3.8 Quality assessment (risk of bias)

To assess the reliability of the qualifying clinical studies, two independent reviewers (MS and EZ) conducted a rigorous and dependable quality assessment using the Cochrane Risk of Bias 2 tool, which has been specifically designed for both parallel and crossover designs. Following evaluation of included studies, any discrepancies between MS and EZ were considered in order to reach a consensus. In cases where consensus was difficult to achieve, a consultation with the third author (AC) was sought to make the final decision. Visual inspection of funnel plots was used to identify potential publication bias.

### 3.9 Certainty of evidence

The level of evidence certainty was evaluated utilizing the GRADE (Grades of Recommendation, Assessment, Development and Evaluation) system through GRADEpro GDT: GRADEpro Guideline Development Tool [Software], McMaster University and Evidence Prime, 2023 (Accessible at gradepro.org).

### 3.10 Meta‐analysis calculations

To assess effect size, we make use of the mean difference (MD) for those studies in which the units and biological materials used for concentration measurements were consistent. In other instances, we used the Cohen's *d* effect size for the standardized mean differences (SMD).

Descriptive analyses were conducted using Microsoft Excel, while meta‐analysis statistics were calculated using Review Manager (RevMan) version 5.4 (Nordic Cochrane Centre, Cochrane Collaboration, Copenhagen, 2014). MD or SMD, the number of participants, and the standard error of SMD were used for each study to quantitatively depict changes in oxidative, immunological and muscle damage biomarkers, when comparing NAC consumption with the placebo group.

A random‐effects model was employed on account of potential variation in the effects of NAC in terms of dosage and other participant‐related moderators. Cohen's criteria were used to interpret the size of SMD: <0.2 was taken to be negligible, 0.2–0.5 as small, 0.5–0.8 as moderate, and over 0.8 as large.^25^ To assess heterogeneity, the *I* ^2^ statistic was calculated; this indicates the percentage of observed overall variability between the studies due to true heterogeneity. An *I* ^2^ value between 25% and 50% signifies low inconsistency, between 50% and 75% indicates moderate heterogeneity, and an *I* ^2^ value exceeding 75% suggests substantial heterogeneity.^26^

## 4 RESULTS

**FIGURE 1:**
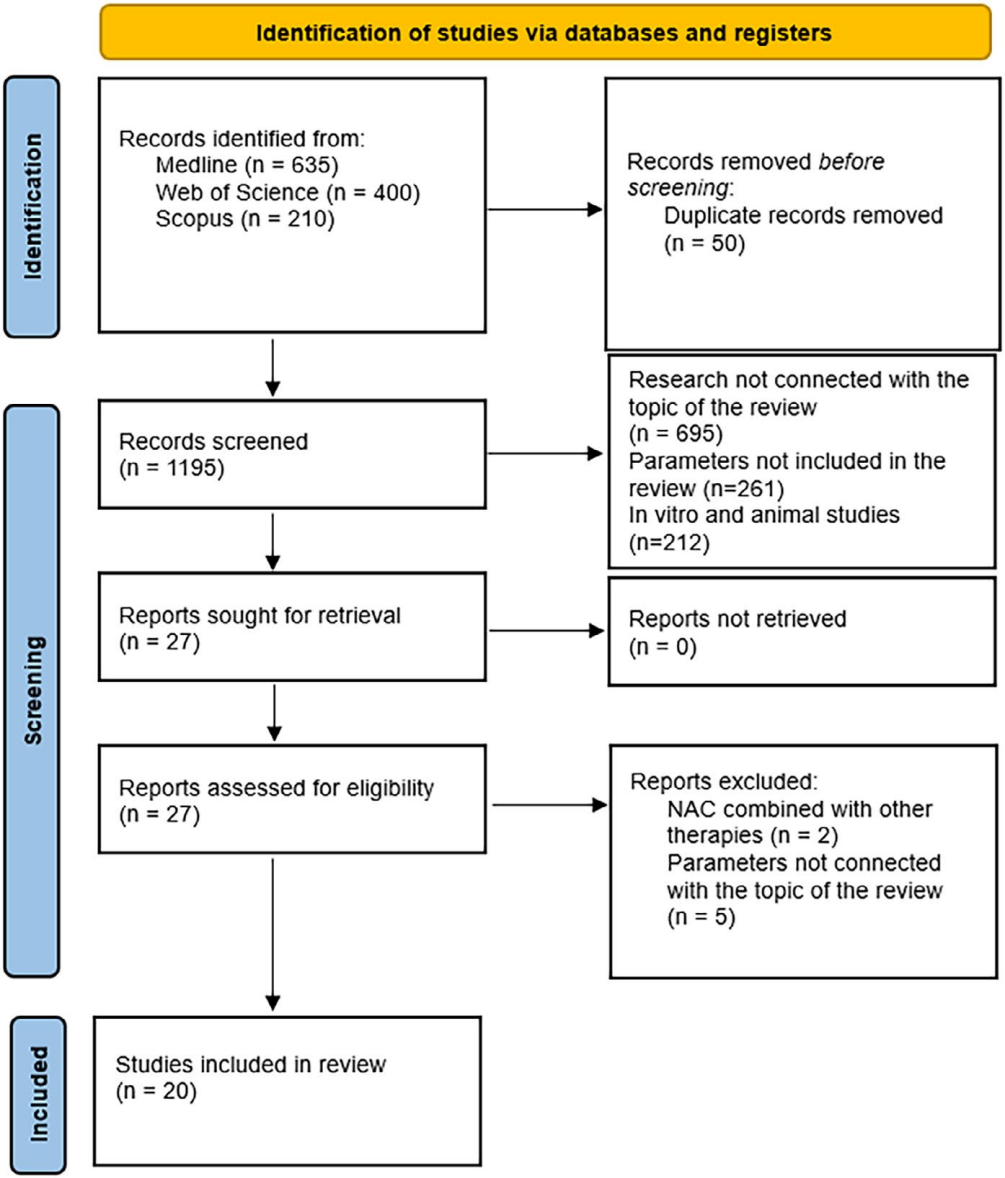
Prisma 2020 flow diagram.

Using our systematic search strategy, a total of 1245 studies were identified, 635 in Medline, 400 in Web of Science and 210 in Scopus. After removing duplicates, this initial pool was reduced to 1195 studies. Subsequently, 1168 studies were excluded during the abstract review phase for not meeting the predefined inclusion criteria. Of the remaining 27 studies, seven were additionally excluded because of the established exclusion criteria. Specifically, two studies involved NAC in combination with other therapies and five studies assessed parameters not included in this analysis.^27^, ^28^, ^29^, ^30^, ^31^, ^32^ As a result, a total of 20 studies met the inclusion criteria and were considered to qualify for inclusion in the systematic review.^2^, ^3^, ^11^, ^12^, ^13^, ^14^, ^33^, ^34^, ^35^, ^36^, ^37^, ^38^, ^39^, ^40^, ^41^, ^42^, ^43^, ^44^, ^45^, ^46^ The entire process of study selection is illustrated in the PRISMA 2020 flow diagram (Figure 1).

### 4.1 Participants and general study characteristics

Twenty studies were included in the review. Four studies did not provide information on height.^37^, ^40^, ^43^, ^45^ Additionally, one study omitted information on body weight.^37^ Detailed information about participant and study characteristics are presented in Tables 1 and 2.

**TABLE 1:** Study characteristics.

Study characteristics			
Study	Country	Study design	Sample size
Bailey et al.^33^	United Kingdom	A double‐blind, cross‐over design	8
Christensen et al.^34^	Denmark	A double‐blinded crossover design	11
Cobley et al.^11^	United Kingdom	A pair‐matched design	12
Ferreira et al.^35^	United States	A double‐blinded, placebo‐controlled, crossover design	17
Kelly et al.^36^	United States	A randomized, double blind cross‐overstudy design	9
Kerksick et al.^13^	United States	A double blind, placebo controlled, parallel study design	30
Leelarungrayub et al.^37^	Thailand	A randomized controlled study design	29
Medved et al.^38^	Australia	A double‐blind, randomized, crossover design	8
Merry et al.^39^	Australia	A double‐blind randomized cross‐over design	9
Michailidis et al.^14^	Greece	A double‐blind, crossover design	10
Moraes et al.^40^	Brazil	A placebo‐controlled study design	20
Nielsen et al.^41^	Denmark	A double‐blinded randomized placebo controlled design	19
Paschalis et al.^3^	Greece	A double‐blind cross‐over design	36
Rhodes et al.^47^	New Zealand	A double‐blind, pre‐post controlled trial design	17
Sakelliou et al.^12^	Greece	A two‐trial, double‐blind, crossover, repeated measures design	10
Sen et al.^43^	Finland	A before and after study design	9
Silva et al.^2^	Brazil	A single‐blind, placebo‐controlled study design	29
Slattery et al.^44^	Australia	A double‐blind randomized placebo‐controlled crossover design	8
Trewin et al.^45^	Australia	A double‐blind, repeated‐measures, randomized crossover trial	9
Trewin et al.^46^	Australia	A double‐blind, randomized, repeated‐measures, crossover design	7

**TABLE 2:** Participants characteristics.

Participant characteristics				
Study	Age [years]	Height [cm]	Body weight [kg]	Training level
Bailey et al.^33^	27 ± 8	180 ± 2	80 ± 7	Recreationally trained men
Christensen et al.^34^	27 ± 8	183 ± 7	73 ± 10	Cyclists
Cobley et al.^11^	27.7 ± 4.2	172.1 ± 4.9	70.1 ± 6.9	Recreationally trained men
Ferreira et al.^35^	30 ± 2	n/a	86 ± 5	Healthy untrained men
Kelly et al.^36^	22.2 ± 2.3	181.1 ± 11.1	77.3 ± 15.7	Recreationally trained healthy men
Kerksick et al.^13^	20 ± 1.8	160 ± 7.1	76.1 ± 17	Active. non‐resistance trained men
Leelarungrayub et al.^37^	19.49	n/a	n/a	Healthy untrained men
Medved et al.^37^	27.sty	180.3 ± 5.4	76.7 ± 10.9	Endurance male
Merry et al.^39^	23	179 ± 3	79.7 ± 3	Recreationally trained healthy men
Michailidis et al.^14^	23.maj	177 ± 10	76.3 ± 9	Recreationally trained healthy men
Moraes et al.^40^	15	n/a	74.2 ±	Male volleyball
Nielsen et al.^41^	27	189 ± 2	82 ± 2	Trained male oarsmen
Paschalis et al.^3^	23.49	177 ± 5.37	73.34 ± 7.78	Recreationally trained young men
Rhodes et al.^47^	20.4 ± 0.9	182.3 ± 7.4	103 ± 12	Semi‐elite male rugby players
Sakelliou et al.^12^	24.2 ± 2.1	181 ± 10	78.5 ± 7.8	Young men
Sen et al.^43^	30 ± 1.7	n/a	76.13 ± 3.37	Active men
Silva et al.^2^	21.3 ± 4	177.2	74.5 ± 7.7	Healthy untrained men
Slattery et al.^44^	23.6 ± 3.2	179.8	70.5 ± 7.2	Well trained male triathletes
Trewin et al.^45^	28 ± 6	n/a	75.6 ± 6.5	Cyclists
Trewin et al.^46^	22.1 ± 3.2	180 ± 10	81.1 ± 14.1	Healthy untrained men

### 4.2 Intervention characteristics

This systematic review extracted data related to substance concentration in response to NAC supplementation and placebo. In the case of the study of Kerksick et al. only data on NAC and placebo supplementation were utilized, with the EGCG data being excluded.^13^ Consequently, an equal number of individuals were assumed to be assigned to each study group. In the case of three parallel‐group studies, the number of individuals in the experimental and control groups differed.^2^, ^33^, ^47^ The total number of individuals in the parallel‐group studies was thus 54 in the control group and 57 in the experimental group. Additionally, Kerksick et al. did not provide information on the allocation of individuals to the control and experimental groups in their study.^13^

Studies were included in our analysis regardless of the administered dose and mode of administration. NAC was administered orally (in five studies) and intravenously (in 15 studies). Both once‐off and continuous supplementation was used. NAC doses were presented in terms of amount per kilogram of participant body weight and as daily dose. Three studies employed acute supplementation, which involved intravenously administering NAC at a rate of 125 mg/kg body weight per hour for 15 min prior to exercise, followed by 25 mg/kg body weight per hour during exercise.^33^, ^38^, ^39^ Trewin et al. also employed a loading phase before the exercise, using an initial dose reduced from 125 mg/kg body weight for 15 min to 62.5 mg/kg/h for 15 min.^46^ Subsequently, continuous supplementation was performed with injections of 25 mg/kg body weight per hour for 80 min. Ferreira et al. used two protocols, each involving supplementation 1 day prior to exercise. In the first protocol, participants received two capsules, each containing 300 or 600 mg NAC. In total, the participants took 600 or 1200 mg NAC, equivalent to 9 and 18 mg/kg body weight. The second protocol involved intravenous supplementation in doses of 35, 70, and 140 mg/kg body weight.^35^ The study of Christensen et al. used oral supplementation just before exercise at 20 mg/kg body weight.^34^ NAC doses in the last two studies involving acute supplementation ranged from 500 mg in five doses of 100 mg to 1800 mg in a single dose.^36^, ^45^

Intervention durations with continuous supplementation ranged from 3 to 30 days. The mean duration of supplementation was 6.2 ± 7.3 days, with a median of 3 days. Four of the studies divided doses of 1200 mg into two equal portions of 600 mg each.^3^, ^37^, ^40^, ^44^ In the remaining publications, the daily doses were 10 mg/kg body weight,^2^ 20 mg/kg body weight,^12^, ^14^ 2 × 50 mg/kg body weight,^11^ 800 mg (4 × 200 mg),^43^ 1000 mg,^47^ 1800 mg^13^ or 6 g.^41^ More details about intervention characteristics can be found in Table 3.

**TABLE 3:** Intervention characteristics.

Intervention characteristics				
Study	Dose	Form of application	Duration	Performance test
Bailey et al.^33^	125 mg/kg b.w./h for15 min before exercise, 25 mg/kg b.w./h throughout exercise	Infusion	Acute	A series of “step” cycle exercise tests that comprised two bouts of moderate‐intensity exercise and one bout of severe intensity exercise
Christensen et al.^34^	20 mg/kg b.w.	Oral	Acute: before exercise	4 min test + 90 min + 4 min test
Cobley et al.^11^	2 × 50 mg/kg b.w.	Oral	Chronic: 6 d and single dose before exercise	A pre‐exercise IKD test, a damaging intermittent‐exercise protocol, YIRT‐L1, and post‐exercise IKD test
Ferreira et al.^35^	Study 1: NAC (2 × 300 or 2 × 600 mg) (9 or 18 mg/kg b.w.) or placebo on the day before exercise	Oral	Acute	A bout of fatiguing handgrip exercise: a bout of fatiguing handgrip exercise MVC using three 5‐s efforts at 30‐s intervals, until the subject failed to reach the target force (70% MVC), followed by a sequence of repetitive isometric manoeuvres.
	Study 2: NAC (35, 70, or 140 mg/kg b.w.) or placebo on the day before exercise			
Kelly et al.^36^	1800 mg/day	Oral	Acute: 45 min before exercise	Two 30 min constant load (85% VO2peak), discontinuous exercises
Kerksick et al.^13^	1800 mg/day	Oral	Chronic: 14 days	One eccentric exercise bout (100 repetitions at 30/s) using the dominant knee extensors
Leelarungrayub et al.^37^	2 × 600 mg/day	Oral	Chronic: 7 days	A graded exercise treadmill test before and after supplementation
Medved et al.^37^	125 mg/kg b.w./h for15 min, 25 mg/kg/h for 20 min prior to and throughout exercise	Infusion	Acute: before and during exercise	A submaximal ergometer cycling for 45 min at 71%VO2max, then continued at 92% until exhaustion
Merry et al.^39^	125 mg/kg/h for15 min, then 25 mg/kg/h for20 min prior to and throughout exercise	Infusion	Acute	An ergometer cycling for 80 min at 62% ± 1% peak oxygen consumption (VO2peak)
Michailidis et al.^14^	20 mg/kg b.w.	Oral	Chronic: during 7 days after training	A muscle‐damaging exercise, comprised 300 eccentric contractions
Moraes et al.^40^	2 × 600 mg/day	Oral	Chronic: 7 days	60 min of volleyball training +30 min running + strength endurance
Nielsen et al.^41^	6 g/day	Oral	Chronic: 3 days before exercise	6 min rowing
Paschalis et al.^3^	2 × 600 mg/day	Oral	Chronic: 30 days	VO2max, Wingate test, time trial cycling for 45 min with 60 rpm at 70% Wmax and subsequently performing as much work as possible for 15 min
Rhodes et al.^47^	1 g/day	Oral	Chronic: 6 days	A broken bronco exercise test
Sakelliou et al.^12^	20 mg/kg/day	Oral	Chronic: 8 days	A muscle‐damaging exercise protocol
Sen et al.^43^	4 × 200 mg/day	Oral	Chronic: 3 days	Cycling exercises for 30 min at aerobic and anaerobic thresholds Maximal bicycle ergometer exercise
Silva et al.^2^	NAC (10 mg/kg b.w.) (oral) for 21 days or NAC plus placebo (14 day NAC + 7 day placebo) or placebo, for14 days before exercise and for 7 days post‐exercise	Oral	Chronic	Eccentric exercise (3 sets until exhaustion of elbow flexion and extension on the Scott bench, 80% 1RM)
Slattery et al.^44^	2 × 600 mg/day	Oral	Chronic: 9 days	105‐min fatigue‐inducing cycle protocol
Trewin et al.^45^	5 × 100 mg/kg	Oral	Acute: before exercise	6 × 5 min HIIE bouts at 82% PPO (316 ± 40 W) separated by 1 min at 100 W, and then after 2 min of recovery at 100 W, TT 10
Trewin et al.^46^	Initial loading dose of 62.5 mg/kg/h for the first15 min, followed by a constant infusion of 25 mg/kg/h for the next 80 min	Infusion	Acute	One hour cycling exercise sessions 7–14 days apart, (55 min at 65% VO2peak plus5 min at 85% VO2peak)

Abbreviations: HIIE, high intensity interval exercise; NAC, N‐acetylcysteine; PPO, peak power output; TT, time trial.

### 4.3 Quality assessment

The results of the quality assessment are provided in the Appendix S2. The risk assessment in the crossover studies points doubts about the quality in four studies, while the risk of bias was considered low in the remaining 10. The analysis of bias risk in parallel‐group studies showed some concerns about bias in four studies, whereas the risk of bias was considered low in two studies.

### 4.4 Certainty of evidence

The summary table of certainty of evidence is available in Appendix S3. Two outcomes (TBARS, IL‐6) were rated as “high,” one outcome (lactate) was rated as “moderate,” three outcomes (muscle soreness–total, muscle soreness‐immediately after exercise and Muscle soreness‐24 h after exercise) as “low” certainty and six of the included outcomes (GSH, GSSG, TNF‐α, CK 2‐6 h after exercise, CK 24 h after exercise, CK 48 h after exercise) were assessed as very low certainty.

### 4.5 Performance tests

The meta‐analysis incorporated studies employing diverse exercise tests to assess the effects of NAC supplementation. Each study selected a distinct exercise protocol to evaluate the physiological responses of participants. In the study of Bailey et al. a test involving a series of exercise sessions on a cycle ergometer was employed, consisting of two sets of moderate‐intensity exercises and one set of high‐intensity exercises.^33^ Christensen et al. utilized a training protocol involving two exercise tests on a cycle ergometer with a 90‐min break between them. Each test lasted for 4 min, with the second test asking participants to exert their maximum effort, while the details of the modification applied to the first test were not described.^34^ Cobley et al. used a series of tests, the first one being an isokinetic dynamometry (IKD) test involving three repetitions at three different speeds using the Biodex system.^11^ After completing the first stage, the participants proceeded to the 60‐min Loughborough Intermittent Shuttle Test. This test includes successive stages in the following order: a sprint at 95% maximal oxygen uptake (VO_2_max) intensity (60 m), jogging at 55% VO_2_max intensity (60 m), walking (60 m), and a maximal sprint (20 m). The next stage of the protocol involved participants in the Yo‐Yo Intermittent Recovery Test Level 1 test, which consists of repeated sprints over a distance of 2 × 20 m with a 10‐s break, increasing the sprint speed each time (0.5 km/h). The protocol concluded with a repeat of the IKD test. The study of Ferreira et al. employed a dynamometer test.^35^ The protocol involved three maximal contractions lasting 5 s each, separated by a 1‐min break, followed by a series of isometric contractions with a three‐second contraction and a three‐second rest sequence. The test was terminated when the participant could not generate a force equal to 70% of the maximal contraction in three consecutive attempts. Kelly et al. employed 30‐min constant load discontinuous trials at 85% VO_2_peak.^36^ Other studies utilized tests such as performing 100 eccentric contractions of the lower limb muscles,^13^ a progressive treadmill test,^37^ stationary cycling at 71% VO_2_max for 45 min followed by 92% VO_2_max until exhaustion,^38^ cycling on a stationary bike for 80 min at 64% VO_2_peak intensity,^39^ performing 300 eccentric contractions using the Biodex system,^14^ and a 60‐min volleyball‐specific training session combined with a 30‐min running session, concluding with endurance strength training.^40^ Detailed information about each performance test used in included studies can be found in Table 3.

### 4.6 Quantitative analysis of primary outcomes

#### 4.6.1 GSH and GSSG concentrations

**FIGURE 2:**
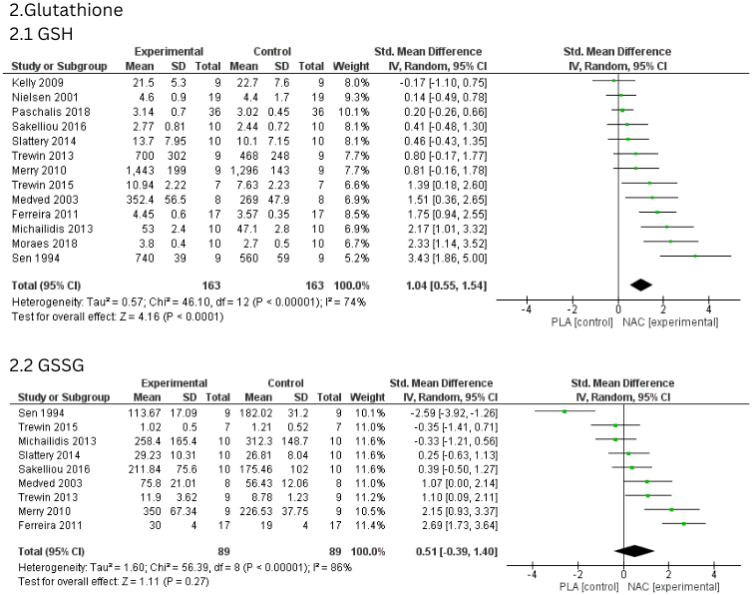
(2.1) Effects of N‐acetylcysteine on the level of reduced glutathione, (2.2) Effects of N‐acetylcysteine on the level of the oxidized form of glutathione.

Data from 13 studies were analysed for changes in GSH concentration after NAC supplementation.^3^, ^12^, ^14^, ^35^, ^36^, ^38^, ^39^, ^40^, ^41^, ^43^, ^44^, ^45^, ^46^ The SMD for the effects of NAC on GSH concentration was 1.04 (95% CI, 0.55, 1.54, medium heterogeneity, *I* ^2^ = 74%), indicating that NAC had a strong effect on the concentration of this parameter (Figure 2). Our statistical analysis demonstrated a significant increase (*p* < 0.00001) in GSH level after NAC supplementation. After conducting sensitivity analysis, no significant changes in the results were observed.

Nine studies were included in our analysis of GSSG concentration, which did not reveal any significant impact (*p* = 0.27; SMD = 0.51, 95% CI, −0.39, 1.40) (Figure 2).^12^, ^14^, ^35^, ^38^, ^39^, ^43^, ^44^, ^45^, ^46^ Analysis of heterogeneity of included studies showed high heterogeneity (*I* ^2^ = 86%, *p* < 0.00001). The sensitivity analysis revealed that after excluding the results from the study by Sen et al.^43^ GSSG levels significantly increased following NAC supplementation (*p* = 0.03; SMD = 0.085, 95% CI, 0.009, 1.61).

#### 4.6.2 TBARS concentration

**FIGURE 3:**
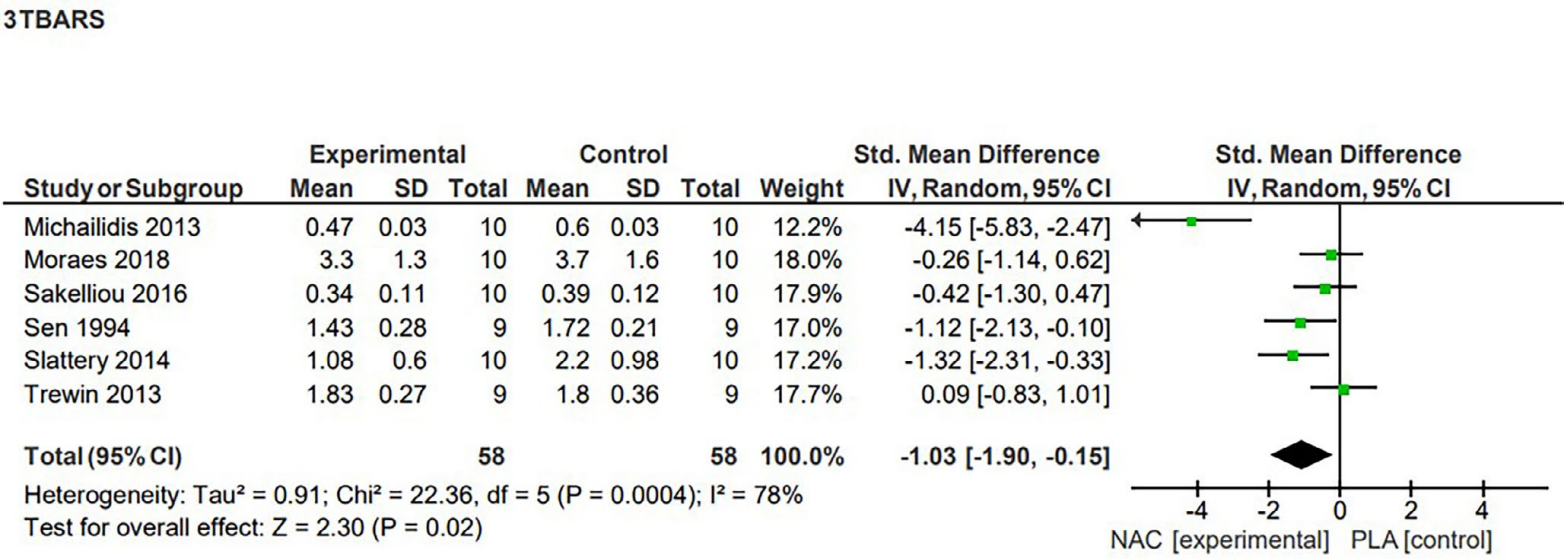
Effects of N‐acetylcysteine on the level of TBARS.

Our statistical analysis of the six studies that investigated the effects of NAC supplementation on TBARS concentration showed a significant decrease (*p* = 0.02) in this marker of lipid peroxidation after supplementation.^12^, ^14^, ^40^, ^43^, ^44^, ^45^ This effect, represented by a reduction in TBARS concentration after exercise, was characterized as large, however the analysis of heterogeneity showed the included studies to be highly diverse (SMD = −1.03, 95% CI, −1.90, −0.15, high heterogeneity, *I* ^2^ = 78%) (Figure 3). The sensitivity analysis indicated that excluding results from both the study by Sen et al.^43^ (*p* = 0.06, SMD = −1.04, 95% CI, −2.10, 0.02) and Slattery et al.^44^ (p = 0.06, SMD = −1.03, 95% CI, −1.90, 0.15) lead to a loss of statistical significance.

#### 4.6.3 IL‐6 concentration

**FIGURE 4:**
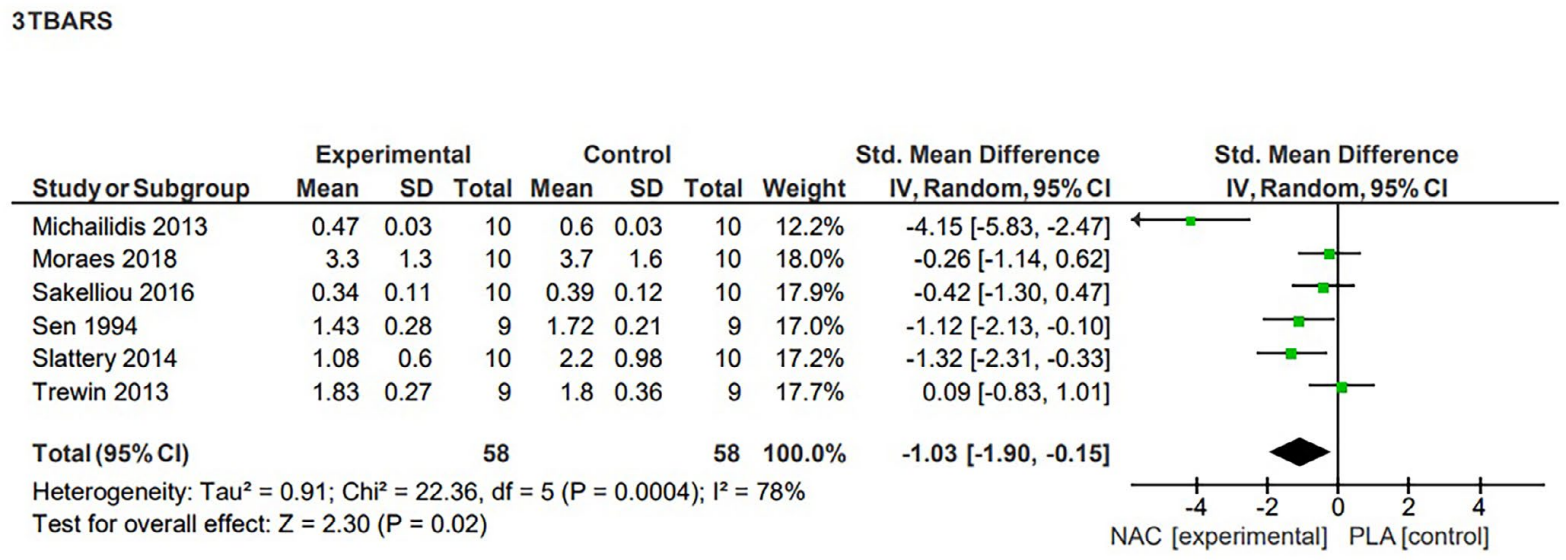
Effects of N‐acetylcysteine on IL‐6 concentration after exercise.

The SMD for the impact of NAC on IL‐6 concentration post‐exercise was −1.71 (95% CI, −3.26, −0.16), indicating a large effect size (SMD> 0.8). Furthermore the analysis of four studies^12^, ^14^, ^44^, ^46^ revealed a significant (*p* = 0.03) reduction in IL‐6 concentration after exercise with NAC supplementation, despite substantial heterogeneity (*I* ^2^ = 86%) (Figure 4). Sensitivity analysis showed that excluding one particular study^14^ alters the heterogeneity from large (*I* ^2^ = 86%, *p* < 0.0001) to none (*I* ^2^ = 0%, *p* = 0.8). Nonetheless the effect of NAC supplementation on IL‐6 after the exclusion of this study decreased from large to moderate (SMD = −0.70, 95% CI,−1.25, −0.14). Excluding results from the study by Sakelliou et al.^12^ (*p* = 0.06, SMD = −2.30, 95% CI, 4.72, 0.12) and Slattery et al.^44^ (*p* = 0.07, SMD = −2.22, 95% CI, −4.65, 0.22) resulted in a loss of statistical significance regarding the effect of NAC supplementation on reducing IL‐6 levels.

#### 4.6.4 TNF‐α concentration

**FIGURE 5:**
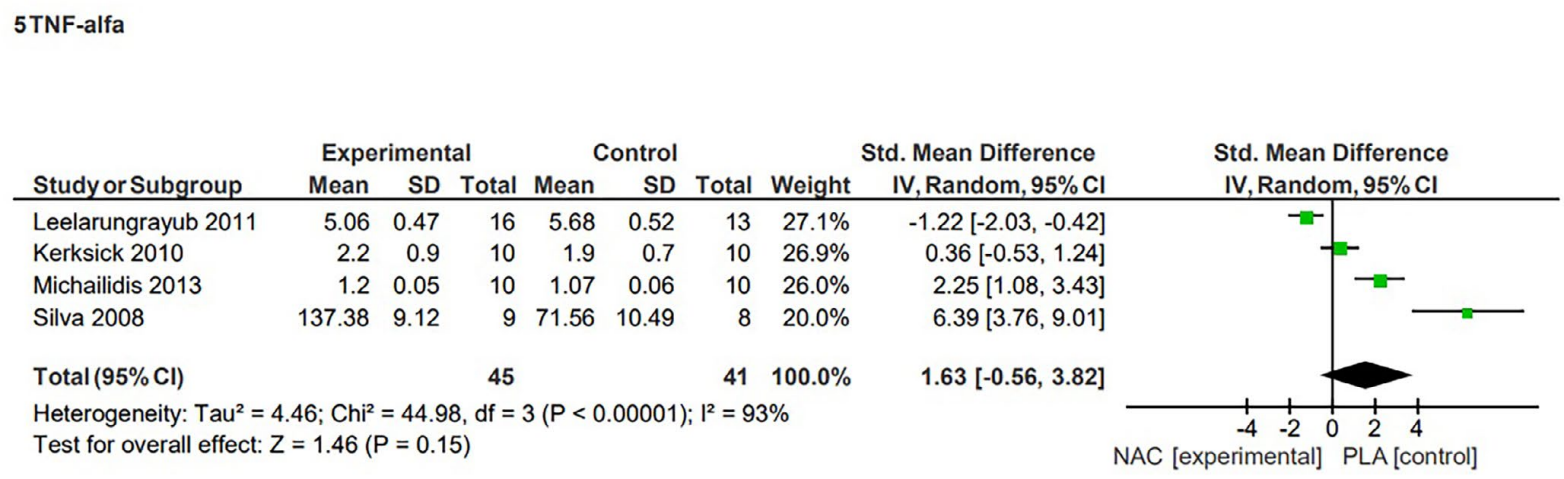
Effects of N‐acetylcysteine on TNF‐α concentration after exercise.

The SMD for the effect of NAC supplementation on TNF‐α concentration post‐exercise showed there to be no significant effect (*p* = 0.15, SMD = 1.63, 95% CI, −0.56, 3.82) with the heterogeneity of the results being assessed as high (*I* ^2^ = 93%, *p* < 0.00001) (Figure 5). The sensitivity analysis demonstrated that excluding the study by Leelarungrayub et al.^37^ resulted in achieving statistical significance (*p* = 0.04, SMD = 2.69, 95% CI, 0.13, 5.24) concerning the impact of NAC supplementation on post‐exercise TNF‐α levels.

#### 4.6.5 Muscle soreness

The quantitative assessment of the effects of NAC supplementation and placebo on exercise‐induced muscle soreness included data from six studies.^2^, ^11^, ^12^, ^13^, ^14^, ^47^ Evaluation of these selected studies involved the measurements of muscle soreness at various time points (immediately after exercise, and 24 h after). Data from five studies were included for the immediate post‐exercise measurement,^11^, ^12^, ^13^, ^14^, ^47^ while data from six studies were included for muscle soreness measured 24 h after exercise cessation.^2^, ^11^, ^12^, ^13^, ^14^, ^47^

**FIGURE 6:**
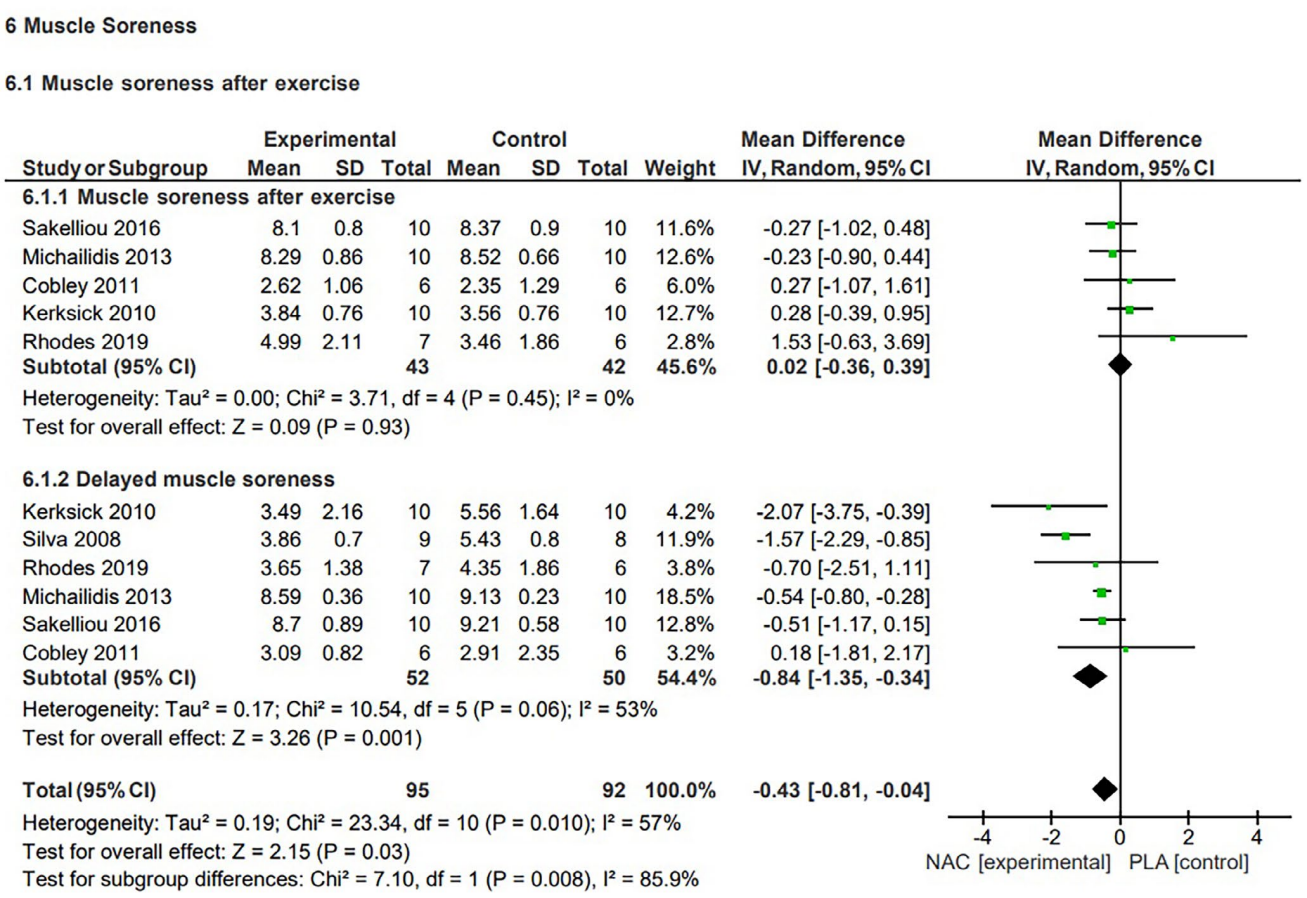
Effects of N‐acetylcysteine on subjective muscle soreness after exercise.

The analysis revealed that NAC significantly (*p* = 0.03) reduced muscle soreness after exercise, regardless of the time. The MD for NAC's effect on muscle soreness was −0.43 (95% Confidence Interval (CI), −0.81, −0.04), with a moderate heterogeneity of *I* ^2^ = 57%. Subgroup analysis demonstrated a significant reduction in muscle soreness 24 h after exercise (MD = −0.84, 95% CI, −1.35, −0.34, *p* = 0.001, medium heterogeneity *I* ^2^ = 52%). However, there was no statistically significant reduction (MD = 0.02, 95% CI, −0.36, 0.39, *p* = 0.93, low heterogeneity *I* ^2^ = 0%) in perceived muscle soreness immediately after exercise connected with NAC supplementation (Figure 6). The sensitivity analysis did not reveal significant changes in the analysed parameter upon exclusion of any of the studies.

#### 4.6.6 Lactate concentration

**FIGURE 7:**
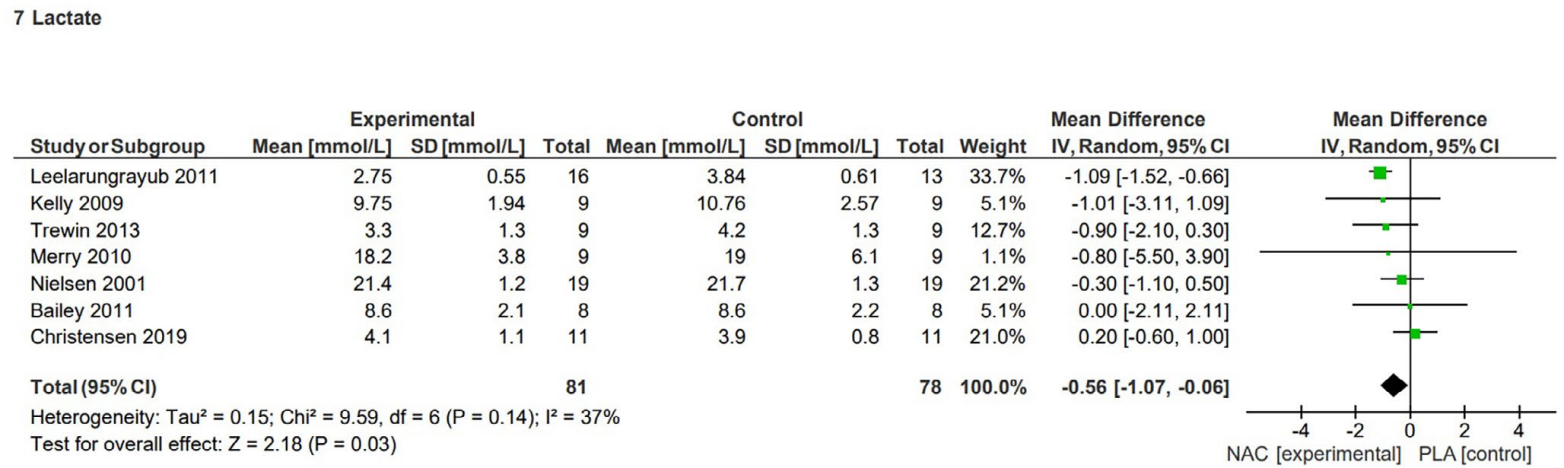
Effects of N‐acetylcysteine on lactate concentration after exercise.

The effect of NAC supplementation on lactate concentration subsequent to exercise was analysed using data from seven studies.^33^, ^34^, ^36^, ^37^, ^39^, ^41^, ^45^ The MD for the effect of NAC on lactate concentration after exercise, compared to placebo, was −0.56 mmol/L (95% CI, −1.07, −0.06), with a low heterogeneity of *I* ^2^ = 37% (*p* = 0.14) among the included studies (Figure 7). The evaluation indicated a significant (*p* = 0.03) reduction in lactate concentration after exercise associated with NAC supplementation (Figure 7). The sensitivity analysis indicated that excluding results from the studies by Kelly et al.^36^ (*p* = 0.07, MD = −0.53, 95% CI, −1.09, 0.04), Leelarungrayub et al.^37^ (*p* = 0.32, MD = −0.24, 95% CI, −0.73, 0.24), and Trewin et al.^45^ (*p* = 0.11, MD = −0.49, 95% CI, −1.10, 0.11) resulted in a loss of statistical significance effect of NAC on the reduction in lactate level.

#### 4.6.7 CK concentration

**FIGURE 8:**
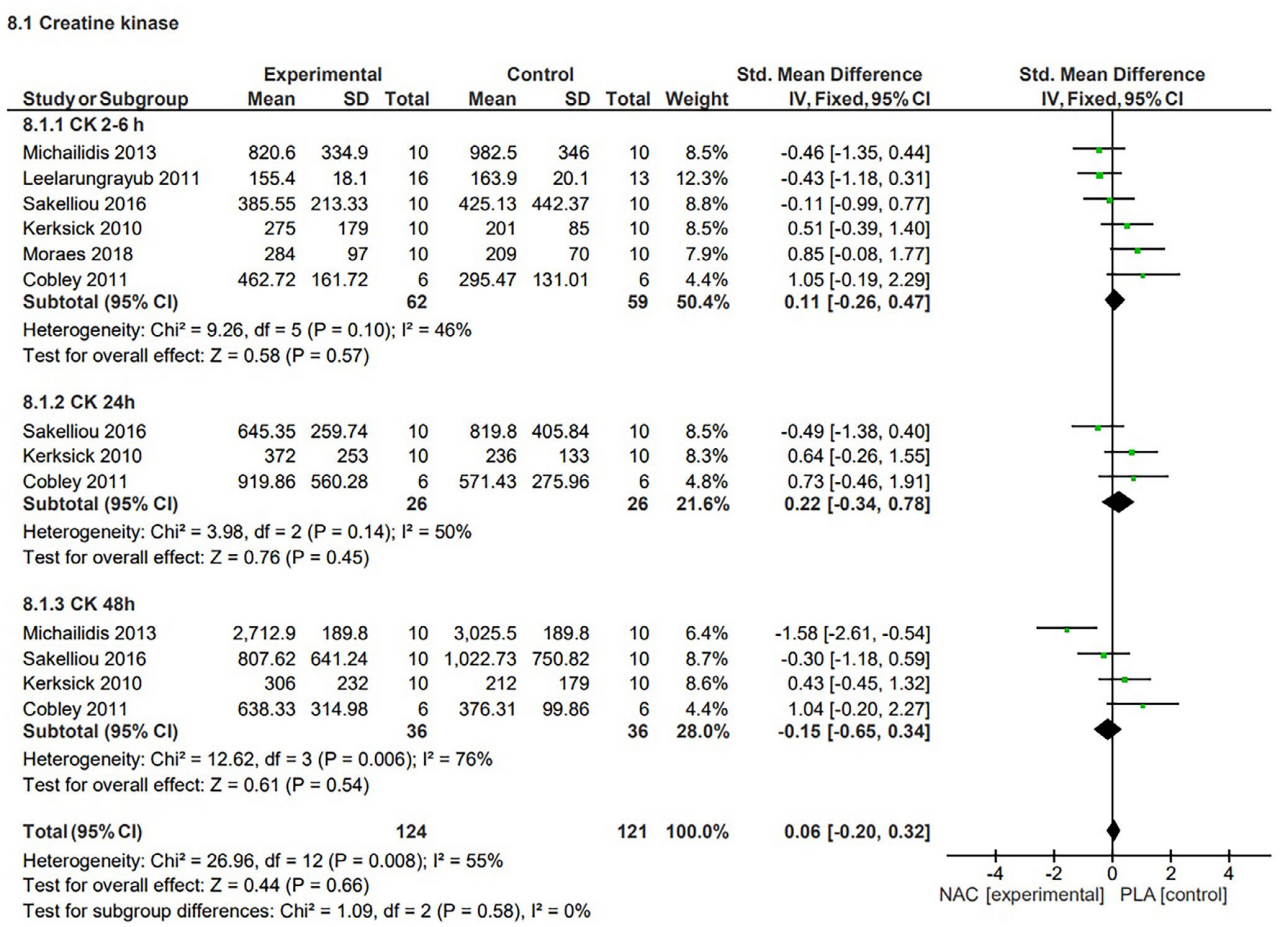
Effects of N‐acetylcysteine on creatine kinase level after exercise.

The studies that assessed the effects of NAC supplementation on CK concentration considered changes in this parameter at several time points. Three time points were included in our analysis: two to 6 h after exercise,^11^, ^12^, ^13^, ^14^, ^37^, ^40^ 24 h after exercise,^11^, ^12^, ^13^ and 48 h after exercise.^11^, ^12^, ^13^, ^14^ Analysis of these three time points across the included studies did not reveal any significant effect of NAC supplementation on CK concentration changes (*p* = 0.63, SMD = 0.10, 95% CI, −0.29, 0.49, medium heterogeneity *I* ^2^ = 55%). Our analysis of the effectiveness of NAC supplementation on CK concentration at different time points also did not show any significant impact of NAC on the concentration of this parameter two to 6 h after exercise (SMD = 0.16, *p* = 0.54, 95% CI; −0.35, 0.67, low heterogeneity, *I* ^2^ = 0.46%), 24 h after exercise (SMD = 0.25, *p* = 0.54, −0.55, 1.05, low/medium heterogeneity, *I* ^2^ = 50%), or 48 hours after exercise (SMD = −0.12, *p* = 0.81, −1.15, 0.90, high heterogeneity, *I* ^2^ = 76%) (Figure 8). The sensitivity analysis did not reveal significant changes in the investigated parameters upon exclusion of individual studies.

## 5 DISCUSSION

As far as we know, this is the first meta‐analysis to focus on the ability of NAC to alter concentrations of biomarkers related to intracellular redox balance, inflammation and muscle damage in response to the specific stimulus of physical exertion. Our study indicates that NAC supplementation significantly diminishes both muscle soreness and lactate concentrations after exercise and decreases concentrations of IL‐6 and TBARS, as well as elevates GSH concentration.

GSH is a major antioxidant that plays a crucial role in the cellular defence against oxidative stress. In this analysis, NAC, as a precursor of GSH, significantly increased GSH concentration, suggesting enhanced antioxidant capacity. GSH—a tripeptide composed of glutamate, cysteine and glycine—serves as substrate for antioxidant enzymes in the first line of defence against oxidative stress by neutralizing ROS and mitigating the harmful effects of oxidative damage.^48^ The significant elevation in GSH level after NAC supplementation suggests that NAC effectively serves as a source of cysteine, thereby supporting the synthesis of GSH. Intense physical activity can lead to the increased generation of ROS which, if left unchecked, may contribute to cellular damage and impair exercise recovery.^8^

In order to maintain cellular integrity, GSH neutralizes ROS, which undergoes oxidation and forms GSSG. Neutralization of ROS thus leads to increased levels of GSSG.^49^ However, in our study the impact of NAC supplementation on GSSG was not statistically significant, indicating potential variations in oxidative stress response. Several factors could contribute to this lack of significance. First, the intricate balance between GSH and GSSG is tightly regulated within cells, and deviations from this balance may not be readily reflected in systemic measurements.^48^ Additionally, the dynamic nature of oxidative stress responses, which are affected by factors such as exercise intensity, duration and individual variability, may contribute to the nuanced results observed in GSSG levels subsequent to supplementation with NAC.^8^, ^50^ An additional important factor that may affect the results is the fact that ex‐vivo oxidation of GSH can be observed during blood collection.^51^

The increase in the concentration of free radicals induced by exercise is associated with increased lipid peroxidation and the accumulation of products of this reaction. Long‐term physical exercise leads to a reduction in the concentration of TBARS in the bloodstream.^52^ However, the reduction in GSH availability noted after intense exercise may be related to increased H_2_O_2_ production, and consequently to increased lipid peroxidation. An increased concentration of lipid peroxidation products is associated with faster occurrence of fatigue during intense exercise.^53^ Our analysis showed a significant reduction in TBARS concentration after exercise associated with NAC supplementation. Taking into account both direct antioxidant properties and indirect properties of NAC related to the increased availability of the substrate for GSH synthesis, NAC effectively neutralizes free radicals and thus contributes to a reduction in the intensity of the lipid peroxidation process. It should however be mentioned that although TBARS has been used for many years, it is nowadays regarded an outdated and non‐specific biomarker of lipid peroxidation. In the studies on the effect of exercise on lipid peroxidation included our analysis TBARS was the only analysed parameter. For this reason it was also shown in the present study, along with GSH and GSSG.

Physical exertion and increased level of free radicals induces an immune response, leading to increased secretion of both proinflammatory and anti‐inflammatory interleukins.^12^ IL‐6 is a pro‐inflammatory cytokine mostly secreted by T‐cells to stimulate the immune response during infection and tissue damage.^10^ Chronic elevated IL‐6 concentration may be associated with atrophy and reduced muscle strength, as well as with a deterioration in the functioning of this tissue.^54^ However, IL‐6 can have both proinflammatory and anti‐inflammatory effects. For example, increased IL‐6 concentration after exercise is associated with increased concentration of anti‐inflammatory IL‐10 and IL‐1RA.^18^, ^55^ Recent research has shown that both NAC and GSH can improve T‐cell function, which can subsequently reduce IL‐6 levels.^1^, ^10^ We observed a significant decrease in IL‐6 concentration after exercise resulting from NAC supplementation. The observed reduction in IL‐6 levels subsequent to exertion in our analysis may thus contribute to an inhibition in the signalling associated with the synthesis of anti‐inflammatory interleukins. However, the studies we reviewed did not examine changes in the level of the anti‐inflammatory interleukins IL‐10, IL‐4, IL‐13 and IL‐1RA, a fact that hinders a comprehensive assessment of NAC supplementation on the immunological response of the body after exercise.

Our meta‐analysis did not reveal changes in TNF‐α concentration after supplementation with NAC. Transcription factor NF‐kβ, which is of crucial importance in the body's response to stress, is activated during exercise, resulting in the upregulation of genes responsible for the synthesis of proinflammatory cytokines such as TNF‐α, IL‐1β and IL‐6, as well as anti‐inflammatory and regulatory factors like IL‐4, IL‐10, IL‐1RA and IL‐13, IL‐15.^56^ Previous publications have indicated an ambiguous relationship between physical exercise and the body's response in the form of interleukin production.^18^ Important factors that should be considered here are the type and duration of exercise, as well as genetic diversity, which is directly related to free radical generation and consequently to the production of interleukins, in particular IL‐6 and TNF‐α.^56^, ^57^, ^58^ Moreover, exercise intensity can affect the production of interleukins, especially TNF‐α.^56^ Our review of the exercise performance tests used in the studies shows high variability in type and intensity of exercise, which may partly explain the difference in the levels of immune response biomarkers. These differences may also be related to the fact that the concentration of interleukins increases temporarily and can be difficult to measure.^56^

It is also worth noting that there were no studies that included other important parameters reflecting the immune response to physical exertion and NAC supplementation—particularly IL1 β, IL‐13, IL‐1RA and IL‐15. Only one of the included studies examined the effects of NAC on the level of IL‐10, and those parameters were therefore not included in the analysis. This underscores the need for studies to analyse a broader range of parameters related to the immune response of the body after physical exertion and NAC supplementation.

Muscle soreness, which is indicative of muscle damage and the time required for recovery are crucial factors in exercise performance. Our meta‐analysis reveals that NAC supplementation may help reduce exercise‐induced muscle soreness. Subgroup analysis showed that the most significant reduction in the subjective feeling of soreness occurred 24 h after exercise, though there was no impact on this sensation immediately after workout. Delayed onset muscle soreness (DOMS) is particularly evident 24 h after exercise. DOMS, assessed on a 10‐point pain scale, is often associated with eccentric effort and unconventional forms of exercise,^59^ and may represent mild type 1b muscle damage.^60^ ROS are generated during lengthening muscle action, which are associated with DOMS. Although there is no direct association between ROS and DOMS, transcription factors regulated by redox balance are necessary for muscle recovery and adaptation to specific types of exercise.^61^ DOMS is normal after certain types of exercise, but its prolonged presence may indicate impaired regeneration.

The increased release of CK into the bloodstream and the consequent elevated CK levels are associated with muscle fibre damage.^56^ CK is recognized as a marker of muscle stress associated with tissue damage. Our analysis did not reveal any significant impact of NAC supplementation on CK concentration changes at various time points. Moreover, we also did not observe any significant changes in CK concentration, regardless of the time point at which the biological material was collected for analysis; this suggests that NAC possesses no ability to reduce muscle cell damage during various types of exercise. It should be noted, however, that 48 h after exercise CK levels increase by about 20%, and that its concentrations 72 h after exercise are about 80% higher than baseline values.^62^ The data used in other studies that have examined the effects of NAC on CK levels referred to 2–6, 24, and 48 h after exercise. Unfortunately, no data from 72 h after exercise were available, so direct comparison was not possible.

Lactate, a byproduct of anaerobic metabolism, has long been associated with fatigue during exercise. However, it plays a crucial role in glucose homeostasis and can be a significant substrate for gluconeogenesis. An increase in exercise intensity is associated with the accumulation of lactate.^50^ Some studies have also indicated that increased lactate levels are also associated with adaptation to endurance exercise.^62^ We identified a significant decrease in lactate concentration upon administration of NAC. This decline in lactate levels may be attributed to the potential inhibitory effect of NAC on glycolytic enzymes. Notably, Pinheiro et al. demonstrated that injections of NAC in rats subjected to electrostimulation resulted in a diminished concentrations of hexokinase and phosphofructokinase in their skeletal muscles.^63^ The observed reduction in glycolytic activity was corroborated by a subsequent decrease in lactate concentrations.^63^

These findings collectively underscore the potential of NAC supplementation to alter various facets of exercise recovery, encompassing inflammation, muscle damage, lactate metabolism and oxidative stress. The effective synthesis of GSH and the decrease in TBARS, IL‐6, and lactate levels following NAC supplementation suggests this can be an effective strategy for improving recovery after strenuous exercise. Furthermore, the decrease in DOMS after NAC supplementation may allow improved performance during consecutive training sessions, high intensity training sessions or competitions with short intervals between them. While our meta‐analysis provides valuable insights, further research is warranted in order to elucidate the underlying mechanisms and to establish optimal dosages and supplementation protocols. Additionally, investigations into diverse exercise modalities and participant populations will contribute to a more nuanced understanding of NAC's applicability to exercise recovery.

## 6 CONCLUSION

In summary, NAC supplementation showed promise in reducing exercise‐induced muscle soreness, IL‐6, lactate concentration and oxidative stress markers. Our findings suggest that NAC could play a role in enhancing post‐exercise recovery. However, these results should be interpreted with caution, especially in the case of reduced lactate and IL‐6 levels as markers of improved recovery after exercise. Given the lack of studies investigating the effects of NAC on other parameters of the immune response and on parameters related to lactate and adaptation to exercise, we could not be certain how supplementation would affect these parameters. It should be noted that the effects of reduced levels of the biomarkers could be beneficial in the context of recovery, the aspect of adaptation to exercise is not important. NAC supplementation should therefore be recommended during tournaments, after intense, long‐term effort, and where recovery between individual training units or starts is a key aspect. However, NAC supplementation does not seem to be indicated or necessary during the macrocycle preparation period. Further research is needed to explore the underlying mechanisms and to establish the optimal supplementation protocols for different exercise modalities and populations. Our results contribute to the evolving understanding of the potential benefits of NAC in exercise‐related muscle damage and recovery.

### 6.1 Limitations

In the authors' assessment, the study exhibits several limitations. The sample size across analysed works was relatively small, with participation predominantly limited to a few individuals. The search methodology was confined to three databases: MEDLINE, Web of Science and Scopus. The cohort of participants encompassed both untrained individuals and those with varying levels of training experience. Owing to the extensive range of exercise tests conducted, a comprehensive analysis of the impact of supplementation based on the nature of physical exercise (aerobic versus anaerobic) was not feasible. The lack of suitable studies made it impossible to examine additional parameters related to the body's immune response to physical exercise. We would also like to note that the greatest increase in parameters was noted 72 h after exercise, but that we do not have comparative data for this time point. For this reason these data were not included in the analysis. Furthermore, the studies incorporated in the analysis exhibited considerable diversity in terms of the biological materials employed for determining concentrations of the evaluated parameters. Nevertheless, our systematic review and meta‐analysis were prepared in accordance with established guidelines. In our meta‐analysis, we performed a certainty analysis of evidence using the GRADE system. We also performed heterogeneity analysis of the included studies. In addition, levels of significance and effect sizes were assessed for each measured parameter. Our meta‐analysis also provides some important results regarding immune and oxidative responses to NAC, and allows assessment of the effect size the NAC supplementation, which allows preparation of practical recommendations. The meta‐analysis also provides some evidence for the use of NAC at different stages of preparation for competition, which is an extremely important issue from a practical point of view.

## AUTHOR CONTRIBUTIONS

**Marcin Sadowski:** Conceptualization (equal); data curation (lead); formal analysis (lead); investigation (equal); methodology (equal); software (lead); visualization (lead); writing – original draft (lead). **Emilia Zawieja:** Conceptualization (equal); formal analysis (supporting); investigation (equal); methodology (equal); writing – review and editing (supporting). **Agata Chmurzynska:** Conceptualization (equal); investigation (equal); methodology (supporting); supervision (lead); writing – review and editing (lead).

## FUNDING INFORMATION

The Open Access cost was financed by the Polish Minister of Science and Higher Education as part of the Strategy of the Poznan University of Life Sciences for 2024–2026 in the field of improving scientific research and development work in priority research areas.

## CONFLICT OF INTEREST STATEMENT

There were no conflict of interests among authors of this review.

## REGISTRATION NUMBER OF THE REVIEW

This systematic review and meta‐analysis has been registered in the PROSPERO registry (CRD42023414276). The full review protocol is available at: https://www.crd.york.ac.uk/PROSPERO/.

## Supporting information


Appendix S1.

Appendix S2.

Appendix S3.

## Data Availability

The data that support the findings of this study are available from the corresponding author upon reasonable request.
