# Supplementary material for: The impact of N‐acetylcysteine on lactate, biomarkers of oxidative stress, immune response, and muscle damage: A systematic review and meta‐analysis
Source: J Cell Mol Med. 2024 Dec 4;28(23):e70198. doi: 10.1111/jcmm.70198 (PMC11617117; doi:10.1111/jcmm.70198)
Supplement: Supplementary file 1 — Appendix S1. [file JCMM-28-e70198-s001.pdf]

Search number,Query,Sort By,Filters,Search Details,Results,Time
16,(acetylcysteine) AND (exercise) AND (lactate),,"Free full text, Full text, Clinical Trial, Randomized Controlled Trial, Humans, English, Female, Male, Adult: 19+ years, Young Adult: 19-24 years, Adult: 19-44 years, Middle Aged + Aged: 45+ years, Middle Aged: 45-64 years, Aged: 65+ years","((""acetylcystein""[All Fields] OR ""acetylcysteine""[MeSH Terms] OR ""acetylcysteine""[All Fields]) AND (""exercise""[MeSH Terms] OR ""exercise""[All Fields] OR ""exercises""[All Fields] OR ""exercise therapy""[MeSH Terms] OR (""exercise""[All Fields] AND ""therapy""[All Fields]) OR ""exercise therapy""[All Fields] OR ""exercising""[All Fields] OR ""exercise s""[All Fields] OR ""exercised""[All Fields] OR ""exerciser""[All Fields] OR ""exercisers""[All Fields]) AND (""lactat""[All Fields] OR ""lactate s""[All Fields] OR ""lactates""[MeSH Terms] OR ""lactates""[All Fields] OR ""lactic
15,(acetylcysteine) AND (exercise) AND (inflammation),,"Free full text, Full text, Clinical Trial, Randomized Controlled Trial, Humans, English, Female, Male, Adult: 19+ years, Young Adult: 19-24 years, Adult: 19-44 years, Middle Aged + Aged: 45+ years, Middle Aged: 45-64 years, Aged: 65+ years","((""acetylcystein""[All Fields] OR ""acetylcysteine""[MeSH Terms] OR ""acetylcysteine""[All Fields]) AND (""exercise""[MeSH Terms] OR ""exercise""[All Fields] OR ""exercises""[All Fields] OR ""exercise therapy""[MeSH Terms] OR (""exercise""[All Fields] AND ""therapy""[All Fields]) OR ""exercise therapy""[All Fields] OR ""exercising""[All Fields] OR ""exercise s""[All Fields] OR ""exercised""[All Fields] OR ""exerciser""[All Fields] OR ""exercisers""[All Fields]) AND (""inflammation""[MeSH Terms] OR ""inflammation""[All Fields] OR ""inflammations""[All Fields] OR ""inflammation
14,(acetylcysteine) AND (aerobic exercise) AND (creatine kinase),,"Free full text, Full text, Clinical Trial, Randomized Controlled Trial, Humans, English, Female, Male, Adult: 19+ years, Young Adult: 19-24 years, Adult: 19-44 years, Middle Aged + Aged: 45+ years, Middle Aged: 45-64 years, Aged: 65+ years","((""acetylcystein""[All Fields] OR ""acetylcysteine""[MeSH Terms] OR ""acetylcysteine""[All Fields]) AND (""exercise""[MeSH Terms] OR ""exercise""[All Fields] OR (""aerobic""[All Fields] AND ""exercise""[All Fields]) OR ""aerobic exercise""[All Fields]) AND (""creatine kinase""[MeSH Terms] OR (""creatine""[All Fields] AND ""kinase""[All Fields]) OR ""creatine kinase""[All Fields])) AND ((ffrft[Filter]) AND (clinicaltrial[Filter] OR
12,(acetylcysteine) AND (exercise) AND (creatine kinase),,"Free full text, Full text, Clinical Trial, Randomized Controlled Trial, Humans, English, Female, Male, Adult: 19+ years, Young Adult: 19-24 years, Adult: 19-44 years, Middle Aged + Aged: 45+ years, Middle Aged: 45-64 years, Aged: 65+ years","((""acetylcystein""[All Fields] OR ""acetylcysteine""[MeSH Terms] OR ""acetylcysteine""[All Fields]) AND (""exercise""[MeSH Terms] OR ""exercise""[All Fields] OR ""exercises""[All Fields] OR ""exercise therapy""[MeSH Terms] OR (""exercise""[All Fields] AND ""therapy""[All Fields]) OR ""exercise therapy""[All Fields] OR ""exercising""[All Fields] OR ""exercise s""[All Fields] OR ""exercised""[All Fields] OR ""exerciser""[All Fields] OR ""exercisers""[All Fields]) AND (""creatine kinase""[MeSH Terms] OR (""creatine""[All Fields] AND ""kinase""[All Fields]) OR ""creatine kinase""[All
11,(acetylcysteine) AND (exercise) AND (IL-13),,"Free full text, Full text, Clinical Trial, Randomized Controlled Trial, Humans, English, Female, Male, Adult: 19+ years, Young Adult: 19-24 years, Adult: 19-44 years, Middle Aged + Aged: 45+ years, Middle Aged: 45-64 years, Aged: 65+ years","((""acetylcystein""[All Fields] OR ""acetylcysteine""[MeSH Terms] OR ""acetylcysteine""[All Fields]) AND (""exercise""[MeSH Terms] OR ""exercise""[All Fields] OR ""exercises""[All Fields] OR ""exercise therapy""[MeSH Terms] OR (""exercise""[All Fields] AND ""therapy""[All Fields]) OR ""exercise therapy""[All Fields] OR ""exercising""[All Fields] OR ""exercise s""[All Fields] OR ""exercised""[All Fields] OR ""exerciser""[All Fields] OR ""exercisers""[All Fields]) AND (""interleukin 13""[MeSH Terms] OR ""interleukin 13""[All Fields] OR ""il 13""[All Fields])) AND ((ffrft[Filter]) AND

10,(acetylcysteine) AND (exercise) AND (IL-15),,"Free full text, Full text, Clinical Trial, Randomized Controlled Trial, Humans, English, Female, Male, Adult: 19+ years, Young Adult: 19-24 years, Adult: 19-44 years, Middle Aged + Aged: 45+ years, Middle Aged: 45-64 years, Aged: 65+ years","((""acetylcystein""[All Fields] OR ""acetylcysteine""[MeSH Terms] OR ""acetylcysteine""[All Fields]) AND (""exercise""[MeSH Terms] OR ""exercise""[All Fields] OR ""exercises""[All Fields] OR ""exercise therapy""[MeSH Terms] OR (""exercise""[All Fields] AND ""therapy""[All Fields]) OR ""exercise therapy""[All Fields] OR ""exercising""[All Fields] OR ""exercise s""[All Fields] OR ""exercised""[All Fields] OR ""exerciser""[All Fields] OR ""exercisers""[All Fields]) AND (""interleukin 15""[MeSH Terms] OR ""interleukin 15""[All Fields] OR ""il 15""[All Fields])) AND ((ffrft[Filter]) AND
9,(acetylcysteine) AND (exercise) AND (reduced glutathione),,"Free full text, Full text, Clinical Trial, Randomized Controlled Trial, Humans, English, Female, Male, Adult: 19+ years, Young Adult: 19-24 years, Adult: 19-44 years, Middle Aged + Aged: 45+ years, Middle Aged: 45-64 years, Aged: 65+ years","((""acetylcystein""[All Fields] OR ""acetylcysteine""[MeSH Terms] OR ""acetylcysteine""[All Fields]) AND (""exercise""[MeSH Terms] OR ""exercise""[All Fields] OR ""exercises""[All Fields] OR ""exercise therapy""[MeSH Terms] OR (""exercise""[All Fields] AND ""therapy""[All Fields]) OR ""exercise therapy""[All Fields] OR ""exercising""[All Fields] OR ""exercise s""[All Fields] OR ""exercised""[All Fields] OR ""exerciser""[All Fields] OR ""exercisers""[All Fields]) AND (""glutathione""[MeSH Terms] OR ""glutathione""[All Fields] OR (""reduced""[All Fields] AND ""glutathione""[All Fields])
8,(acetylcysteine) AND (exercise) AND (IL-1B),,"Free full text, Full text, Clinical Trial, Randomized Controlled Trial, Humans, English, Female, Male, Adult: 19+ years, Young Adult: 19-24 years, Adult: 19-44 years, Middle Aged + Aged: 45+ years, Middle Aged: 45-64 years, Aged: 65+ years","((""acetylcystein""[All Fields] OR ""acetylcysteine""[MeSH Terms] OR ""acetylcysteine""[All Fields]) AND (""exercise""[MeSH Terms] OR ""exercise""[All Fields] OR ""exercises""[All Fields] OR ""exercise therapy""[MeSH Terms] OR (""exercise""[All Fields] AND ""therapy""[All Fields]) OR ""exercise therapy""[All Fields] OR ""exercising""[All Fields] OR ""exercise s""[All Fields] OR ""exercised""[All Fields] OR ""exerciser""[All Fields] OR
6,(acetylcysteine) AND (exercise) AND (oxidative stress),,"Free full text, Full text, Clinical Trial, Randomized Controlled Trial, Humans, English, Female, Male, Adult: 19+ years, Young Adult: 19-24 years, Adult: 19-44 years, Middle Aged + Aged: 45+ years, Middle Aged: 45-64 years, Aged: 65+ years","((""acetylcystein""[All Fields] OR ""acetylcysteine""[MeSH Terms] OR ""acetylcysteine""[All Fields]) AND (""exercise""[MeSH Terms] OR ""exercise""[All Fields] OR ""exercises""[All Fields] OR ""exercise therapy""[MeSH Terms] OR (""exercise""[All Fields] AND ""therapy""[All Fields]) OR ""exercise therapy""[All Fields] OR ""exercising""[All Fields] OR ""exercise s""[All Fields] OR ""exercised""[All Fields] OR ""exerciser""[All Fields] OR ""exercisers""[All Fields]) AND (""oxidative stress""[MeSH Terms] OR (""oxidative""[All Fields] AND ""stress""[All Fields]) OR ""oxidative stress""[All
5,(acetylcysteine) AND (exercise) AND (TNF-alpha),,"Free full text, Full text, Clinical Trial, Randomized Controlled Trial, Humans, English, Female, Male, Adult: 19+ years, Young Adult: 19-24 years, Adult: 19-44 years, Middle Aged + Aged: 45+ years, Middle Aged: 45-64 years, Aged: 65+ years","((""acetylcystein""[All Fields] OR ""acetylcysteine""[MeSH Terms] OR ""acetylcysteine""[All Fields]) AND (""exercise""[MeSH Terms] OR ""exercise""[All Fields] OR ""exercises""[All Fields] OR ""exercise therapy""[MeSH Terms] OR (""exercise""[All Fields] AND ""therapy""[All Fields]) OR ""exercise therapy""[All Fields] OR ""exercising""[All Fields] OR ""exercise s""[All Fields] OR ""exercised""[All Fields] OR ""exerciser""[All Fields] OR ""exercisers""[All Fields]) AND (""tumor necrosis factor alpha""[MeSH Terms] OR (""tumor""[All Fields] AND ""necrosis""[All Fields] AND ""factor
4,(acetylcysteine) AND (exercise) AND (IL-10),,"Free full text, Full text, Clinical Trial, Randomized Controlled Trial, Humans, English, Female, Male, Adult: 19+ years, Young Adult: 19-24 years, Adult: 19-44 years, Middle Aged + Aged: 45+ years, Middle Aged: 45-64 years, Aged: 65+ years","((""acetylcystein""[All Fields] OR ""acetylcysteine""[MeSH Terms] OR ""acetylcysteine""[All Fields]) AND (""exercise""[MeSH Terms] OR ""exercise""[All Fields] OR ""exercises""[All Fields] OR ""exercise therapy""[MeSH Terms] OR (""exercise""[All Fields] AND ""therapy""[All Fields]) OR ""exercise therapy""[All Fields] OR ""exercising""[All Fields] OR ""exercise s""[All Fields] OR ""exercised""[All Fields] OR ""exerciser""[All Fields] OR ""exercisers""[All Fields]) AND (""interleukin 10""[MeSH Terms] OR ""interleukin 10""[All Fields] OR ""il 10""[All Fields])) AND ((ffrft[Filter]) AND

3,(acetylcysteine) AND (exercise) AND (IL-6),,"Free full text, Full text, Clinical Trial, Randomized Controlled Trial, Humans, English, Female, Male, Adult: 19+ years, Young Adult: 19-24 years, Adult: 19-44 years, Middle Aged + Aged: 45+ years, Middle Aged: 45-64 years, Aged: 65+ years","((""acetylcystein""[All Fields] OR ""acetylcysteine""[MeSH Terms] OR ""acetylcysteine""[All Fields]) AND (""exercise""[MeSH Terms] OR ""exercise""[All Fields] OR ""exercises""[All Fields] OR ""exercise therapy""[MeSH Terms] OR (""exercise""[All Fields] AND ""therapy""[All Fields]) OR ""exercise therapy""[All Fields] OR ""exercising""[All Fields] OR ""exercise s""[All Fields] OR ""exercised""[All Fields] OR ""exerciser""[All Fields] OR ""exercisers""[All Fields]) AND (""interleukin 6""[MeSH Terms] OR ""interleukin 6""[All Fields] OR ""il 6""[All Fields])) AND ((ffrft[Filter]) AND
1,((acetylcysteine) AND (exercise)) AND (glutathione),,"Free full text, Full text, Clinical Trial, Randomized Controlled Trial, Humans, English, Female, Male, Adult: 19+ years, Young Adult: 19-24 years, Adult: 19-44 years, Middle Aged + Aged: 45+ years, Middle Aged: 45-64 years, Aged: 65+ years","((""acetylcystein""[All Fields] OR ""acetylcysteine""[MeSH Terms] OR ""acetylcysteine""[All Fields]) AND (""exercise""[MeSH Terms] OR ""exercise""[All Fields] OR ""exercises""[All Fields] OR ""exercise therapy""[MeSH Terms] OR (""exercise""[All Fields] AND ""therapy""[All Fields]) OR ""exercise therapy""[All Fields] OR ""exercising""[All Fields] OR ""exercise s""[All Fields] OR ""exercised""[All Fields] OR ""exerciser""[All Fields] OR ""exercisers""[All Fields]) AND (""glutathionation""[All Fields] OR ""glutathione""[MeSH Terms] OR ""glutathione""[All Fields] OR ""glutathion""[All Fields]
