# Supplementary material for: The impact of N‐acetylcysteine on lactate, biomarkers of oxidative stress, immune response, and muscle damage: A systematic review and meta‐analysis
Source: J Cell Mol Med. 2024 Dec 4;28(23):e70198. doi: 10.1111/jcmm.70198 (PMC11617117; doi:10.1111/jcmm.70198)

Unique ID	D1a	D1b	D2	D3	D4	D5	Overall
Kerksick et.al 2010	!	!	+	+	+	+	!
Cobley et. al 2011	!	+	+	+	+	+	!
Leelarungrayub et. al 2011	+	+	+	+	+	+	+
Moraes et. al 2018	!	+	+	+	+	+	!
Rhodes et. al 2017	+	+	+	+	+	+	+
Silva et. al 2008	+	+	+	+	!	+	!
Bailey et. al 2011	!	+	+	+	+	+	+
Christiansen et. al 2019	!	+	+	+	+	!	+
Ferreira et. al 2011	!	+	!	+	+	+	!
Kelly et.al 2009	+	+	+	+	+	+	+
Merry et.al 2010	+	+	+	+	+	+	+
Michailidis et. al 2013	+	+	+	+	+	+	+
Nielsen et.al 2000	+	+	+	+	+	+	+
Sakelliou et. al 2016	+	+	+	+	!	+	!
Slattery et.al 2014	+	+	+	+	+	+	+
Trewin et.al 2014	+	+	+	+	+	+	+
Trewin et. al 2015	+	+	+	+	+	+	+
Sen et.al 1994	!	+	+	+	+	+	!
Medved et.al 2004	+	!	!	+	+	+	!
Paschalis et.al 2018	!	!	!	+	+	+	!

+ Low risk

! Some concerns

- High risk

D1a	Randomisation process
D1b	Timing of identification or recruitment of participants
D2	Deviations from the intended interventions
D3	Missing outcome data
D4	Measurement of the outcome
D5	Selection of the reported result

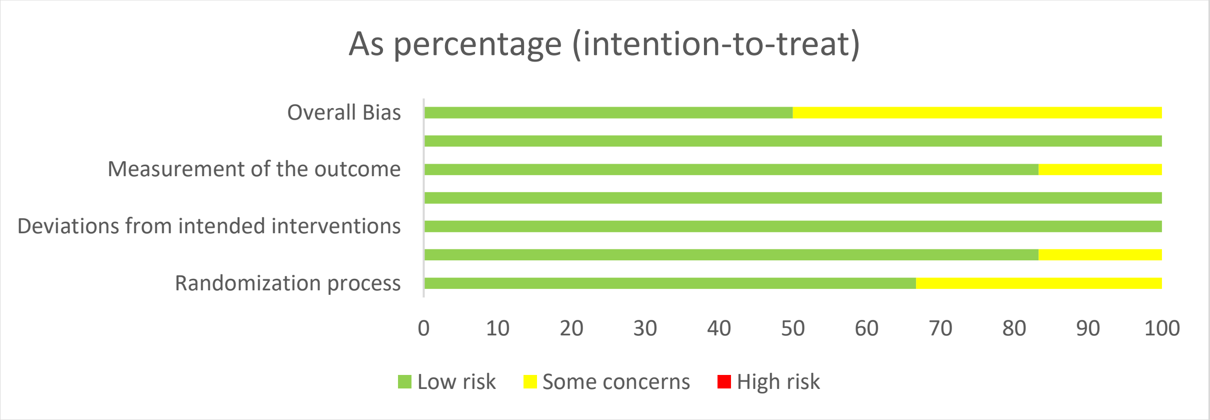

Supplement: Supplementary file 2 — Appendix S2. [file JCMM-28-e70198-s003.pdf]
