# Supplementary material for: The impact of N‐acetylcysteine on lactate, biomarkers of oxidative stress, immune response, and muscle damage: A systematic review and meta‐analysis
Source: J Cell Mol Med. 2024 Dec 4;28(23):e70198. doi: 10.1111/jcmm.70198 (PMC11617117; doi:10.1111/jcmm.70198)
Supplement: Supplementary file 3 — Appendix S3. [file JCMM-28-e70198-s002.pdf]

SUPPLEMENTARY MATERIAL 2

Author(s):
Question: N-acetylocysteine compared to placebo for increasing glutathione level after exercise
Setting:
Bibliography:

Certainty assessment							№ of patients		Effect		Certainty	Importance
№ of studies	Study design	Risk of bias	Inconsistency	Indirectness	Imprecision	Other considerations	N-acetylocysteine	placebo	Relative (95% CI)	Absolute (95% CI)		
**GSH**												
13	non-randomised studies	not serious[a]	serious[b]	not serious	not serious	none	163	163	-	SMD **1.04 SD higher** (0.55 higher to 1.54 higher)	⨁◯◯◯ Very low	CRITICAL
**GSSG**												
9	non-randomised studies	not serious[a]	serious[c]	not serious	not serious	none[a]	89	89	-	SMD **0.51 higher** (0.39 lower to 1.4 higher)	⨁◯◯◯ Very low	CRITICAL

CI: confidence interval; **SMD:** standardised mean difference

Explanations

a. Two of the studies were not randomized, which raises some doubts about the results obtained. In the case of one of the studies, it was a before-and-after study without a control group.
b. There is a large variation in GSH concentrations depending on the study, which is related to differences in the biological material used.
c. There is a large variation in GSSG concentrations depending on the study, which is related to differences in the biological material used.

Author(s):
Question: N-acetylcysteine compared to placebo for reducing TBARS level after exercise
Setting:
Bibliography:

Certainty assessment							№ of patients		Effect		Certainty	Importance
№ of studies	Study design	Risk of bias	Inconsistency	Indirectness	Imprecision	Other considerations	N-acetylcysteine	placebo	Relative (95% CI)	Absolute (95% CI)		
**TBARS**												
6	non-randomised studies	not serious	not serious	not serious	not serious	none	58	58	-	SMD **1.03 SD lower** (1.9 lower to 0.15 lower)	⨁⨁⨁⨁ High	CRITICAL

CI: confidence interval; **SMD:** standardised mean difference

Author(s):
Question: N-acetylcysteine compared to placebo for reducing muscle soreness after exercise
Setting:
Bibliography:

Certainty assessment							№ of patients		Effect		Certainty	Importance
№ of studies	Study design	Risk of bias	Inconsistency	Indirectness	Imprecision	Other considerations	N-acetylcysteine	placebo	Relative (95% CI)	Absolute (95% CI)		
**Muscle soreness - total**												
6	non-randomised studies	not serious	not serious	not serious	not serious	none	95	92	-	MD **0.43 SD lower** (0.81 lower to 0.04 lower)	⊕⊕◯◯ Low	CRITICAL
**Muscle soreness - immediately after exercise**												
5	non-randomised studies	not serious	not serious	not serious	not serious	none	43	42	-	MD **0 SD** (0 to 0)	⊕⊕◯◯ Low	CRITICAL
**Muscle soreness - 24h after exercise**												
6	non-randomised studies	not serious	not serious	not serious	not serious	none	52	50	-	MD **0** (0 to 0)	⊕⊕◯◯ Low	CRITICAL

CI: confidence interval; **MD:** mean difference

Author(s):
Question: N-acetylcysteine compared to placebo for reducing IL-6 level after exercise
Setting:
Bibliography:

Certainty assessment							№ of patients		Effect		Certainty	Importance
№ of studies	Study design	Risk of bias	Inconsistency	Indirectness	Imprecision	Other considerations	N-acetylcysteine	placebo	Relative (95% CI)	Absolute (95% CI)		
**IL-6**												
4	randomised trials	not serious	not serious	not serious	not serious	none	37	37	-	SMD **1.71 lower** (3.26 lower to 0.16 lower)	⊕⊕⊕⊕ High	IMPORTANT

CI: confidence interval; **SMD:** standardised mean difference

Author(s):
Question: N-acetylcysteine compared to placebo for reduce level of TNF-alfa after exercise
Setting:
Bibliography:

Certainty assessment							№ of patients		Effect		Certainty	Importance
№ of studies	Study design	Risk of bias	Inconsistency	Indirectness	Imprecision	Other considerations	N-acetylcysteine	placebo	Relative (95% CI)	Absolute (95% CI)		
**TNF-alfa 24h**												
4	non-randomised studies	not serious	serious[a]	not serious	not serious	none	45	41	-	SMD **1.63 SD higher** (0.56 lower to 3.82 higher)	⨁◯◯◯ Very low	IMPORTANT

CI: confidence interval; **SMD:** standardised mean difference

Explanations

a. The TNF-alpha concentration measured in one of the studies is several dozen times higher than in the other studies included in the analysis.

Author(s):
Question: N-acetylcysteine compared to placebo for decrase level of lactate after exercise
Setting:
Bibliography:

Certainty assessment							№ of patients		Effect		Certainty	Importance
№ of studies	Study design	Risk of bias	Inconsistency	Indirectness	Imprecision	Other considerations	N-acetylcysteine	placebo	Relative (95% CI)	Absolute (95% CI)		
**Lactate**												
7	randomised trials	not serious	serious[a]	not serious	not serious	none	81	78	-	MD **0.56 mmol/L fewer** (1.07 fewer to 0.06 fewer)	⨁⨁⨁◯ Moderate	IMPORTANT

CI: confidence interval; **MD:** mean difference

Explanations

a. There is a large variety of exercise tests in the studies included in the analysis. Therefore, we observe large differences in lactate concentrations depending on the study.

Author(s):
Question: N-acetylcysteine compared to placebo for reduce level of creatine kinase after exercise
Setting:
Bibliography:

Certainty assessment							№ of patients		Effect		Certainty	Importance
**№ of studies**	**Study design**	**Risk of bias**	**Inconsistency**	**Indirectness**	**Imprecision**	**Other considerations**	**N-acetylcysteine**	**placebo**	**Relative (95% CI)**	**Absolute (95% CI)**		
**CK 2 - 6 h after exercise**												
6	non-randomised studies	not serious	serious[a]	not serious	not serious	none	62	59	-	SMD **38.29 SD higher** (27.22 lower to 103.79 higher)	⨁◯◯◯ Very low	IMPORTANT
**CK 24h after exercise**												
3	non-randomised studies	not serious	serious[a]	not serious	not serious	none[a]	26	26	-	SMD **0.25 SD higher** (0.55 lower to 1.05 higher)	⨁◯◯◯ Very low	IMPORTANT
**CK 48h**												
4	non-randomised studies	not serious	serious[a]	not serious	not serious	none	36	36	-	SMD **27.13 SD lower** (325.91 lower to 271.65 higher)	⨁◯◯◯ Very low	IMPORTANT

CI: confidence interval; **SMD:** standardised mean difference

Explanations

a. Very large differences in creatine kinase concentrations after exercise.
